# Regulation of MRTF-A by JMY via a nucleation-independent mechanism

**DOI:** 10.1186/s12964-018-0299-x

**Published:** 2018-11-21

**Authors:** Franziska Kluge, Julia Weissbach, Anja Weber, Theresia Stradal, Guido Posern

**Affiliations:** 1 Institute for Physiological Chemistry, Medical Faculty, Martin Luther University Halle-Wittenberg, 06114 Halle (Saale), Germany; 2 Department of Cell Biology, Helmholtz Centre for Infection Research, 38124 Braunschweig, Germany

**Keywords:** Mrtf, Actin, Transcription, WH2 domains, JMY, Nucleators

## Abstract

**Background:**

MRTF-A (myocardin-related transcription factor A) is a coactivator for SRF-mediated gene expression. The activity of MRTF-A is critically dependent on the dissociation of G-actin from N-terminal RPEL motifs. MRTF-SRF induction often correlates with enhanced polymerization of F-actin. Here we investigate MRTF regulation by the multifunctional JMY protein, which contains three WASP/verprolin homology 2 (WH2/V) domains and facilitates Arp2/3-dependent and -independent actin nucleation.

**Methods:**

Co-immunoprecipitation experiments, immunofluorescence and luciferase reporter assays were combined with selective inhibitors to investigate the effect of JMY and its domains on MRTF-A in NIH 3 T3 mouse fibroblasts.

**Results:**

JMY induced MRTF-A transcriptional activity and enhanced its nuclear translocation. Unexpectedly, MRTF-A was hyperactivated when the Arp2/3-recruiting CA region of JMY was deleted or mutated, suggesting an autoinhibitory mechanism for full-length JMY. Moreover, isolated WH2/V domains which are unable to nucleate actin were sufficient for nuclear accumulation and SRF activation. Recombinant WH2/V regions of JMY biochemically competed with MRTF-A for actin binding. Activation of MRTF-A by JMY was unaffected by Arp3 knockdown, by an Arp2/3 inhibitor, and by latrunculin which disassembles cellular F-actin. Restriction of JMY to the nucleus abrogated its MRTF-A activation. Finally, JMY RNAi reduced basal and stimulated transcriptional activation via MRTF-A.

**Conclusions:**

Our results suggest that JMY activates MRTF-SRF independently of F-actin via WH2/V-mediated competition with the RPEL region for G-actin binding in the cytoplasm. Furthermore, the C-terminal region facilitates an autoinhibitory effect on full-length JMY, possibly by intramolecular folding.

## Background

Regulators of actin polymerization play important roles in determining many aspects of eukaryotic cell behavior, including cell shape, adhesion, vesicular trafficking and migration [1]. Formation of F-actin from monomeric G-actin is catalyzed by actin nucleators such as the Arp2/3 complex, which acts in concert with class I nucleation promoting factors of the WASP superfamily [2]. One member of the WASP family is the multifunctional nucleator JMY (junction-mediating and regulatory protein), which was originally identified as a p53/p300 coactivator [3]. JMY has dual nucleation capabilities: similar to N-WASP and WAVE2, it recruits and activates the Arp2/3 complex via its C-terminal CA (central/connecting, acidic) region [4, 5]. In addition, JMY contains three domains designated WH2/V (WASP/verprolin homology), which facilitate actin nucleation independently of the Arp2/3 complex [5]. The intrinsically disordered WH2/V regions utilize a hydrophobic α-helix and a LKKT/V motif during their conserved mode of binding to the cleft between subdomains 1 and 3 of G-actin [6, 7]. Structural analysis suggested that the arrayed WH2/V domains of JMY are capable of forming a critical tripartite actin nucleation core in the absence of Arp2/3 [8].

Besides its role for the cellular architecture, actin specifically regulates gene transcription via serum response factor (SRF) [9, 10]. The SRF transcriptional coactivator MRTF-A (myocardin-related transcription factor A, syn. MKL1, MAL, BSAC) directly responds to changes in G-actin [11]. The critical regulatory step is the dissociation of MRTF-A from G-actin, which releases MRTF-A from its repressive complex and strongly enhances SRF target gene transcription. G-actin directly interacts with three RPEL motifs and intervening linkers in the N-terminus of MRTF-A [12, 13]. A bipartite nuclear localization signal is embedded within the RPEL domain, preventing importin-dependent nuclear import of the pentameric actin complex [14, 15]. Interestingly, a similar mechanism prevents nuclear import of JMY since G-actin binding occludes a NLS within the WH2 cluster [16]. Correlating with dissociation from G-actin, MRTF-A accumulates in the nucleus by enhanced nuclear import and decreased Crm1-dependent nuclear export [15, 17]. It is thought that induced actin polymerization either in the cytoplasm or in the nucleus reduces the level of G-actin available for MRTF inhibition [18, 19].

Structural analysis revealed that the RPEL motifs adopt a hydrophobic α-helix which, despite an opposite orientation, occupies the same surface in the actin hydrophobic cleft as the WH2 helix [20]. We recently showed that N-WASP and WAVE2 considerably activate MRTF-A via their WH2 domains independently of Arp2/3 [21]. Considering the transcriptional role of JMY via p53 and p300 [3, 4] and its dual actin nucleation capabilities, we now investigated whether JMY activates MRTF-A via nucleation-dependent or -independent mechanisms. We found that JMY augments MRTF-A transcriptional activity and nuclear translocation. Unexpectedly, MRTF-SRF activation was considerably enhanced by deleting or mutating the Arp2/3-recruiting CA region of JMY, indicating that full-length JMY is autoinhibited. Moreover, the threepartite WH2/V array was not required for MRTF-A activation since isolated WH2 domains and tandems were sufficient for nuclear accumulation and SRF activation. Despite different efficiencies, biochemical competition with MRTF-A for actin binding was achieved by recombinant WH2 regions of JMY. Finally, activation of MRTF-A by JMY was unaffected by Arp3 knockdown, by an Arp2/3 inhibitor, and by latrunculin which disassembles cellular F-actin. This F-actin-independent mechanism of MRTF-SRF activation was largely restricted to the cytoplasm, since JMY constructs fused to an NLS were hardly inducing reporter activity.

## Methods

### Plasmids, proteins and reagents

SRF reporters and expression plasmids encoding MRTF-A variants (MRTF-A-f.l., MRTF-A (2–261)), Thymosin β4 or wild-type actin (pEF-Flag-actin-WT) were described previously [9, 11, 19, 21–23]. MRTF-A (2–261) was expressed as GST fusion protein and purified as described [21]. Murine pEGFP-C1-JMY [24] was subcloned into pEF-myc. pEF-myc-NLS-JMY was generated from pEF-myc-JMY by inserting a SV40-derived nuclear localization signal (NLS) (CCG CCT AAG AAA AAG CGG AAG GTG) via PCR. pEF-myc-JMY-∆C (∆945–967), pEF-myc-JMY-R961E and pEF-myc-JMY-∆960–963 were generated by performing Single Oligonucleotide Mutagenesis and Cloning Approach [25]. The truncations pEF-myc-JMY-∆A (1–967), pEF-myc-JMY-∆CA (1–944) and pEF-myc-JMY-∆VCA (1–905) were generated by inserting a premature STOP codon via PCR. pEF-myc-JMY was used to isolate the following V or VVVCA constructs by PCR: V(A, 846–882), V(B, 877–912), V(C, 908–944), VV(AB, 846–912), VV(BC, 877–944), VVV(ABC, 846–944) and VVV(ABC)CA(846–983). Those fragments were inserted into pGEX-6P-1 (GE Healthcare Europe GmbH, Freiburg, Germany) or pEGFP-C2 (Takara Bio Europe S.A.S., Saint-Germain-en-Laye, France). To ensure correct spacing between GST/GFP and V domains, an additional glycine-serine ((GlySer)_5_)-linker was inserted.

Primary antibodies used were anti-GST, anti-GFP, anti-hemagglutinin (anti-HA), anti-Tubulin, anti-Flag (all purchased from Sigma-Aldrich, Steinheim, Germany), anti-MRTF-A (homemade rabbit antiserum [26]), anti-Arp3 (Proteintech Europe, Manchester, United Kingdom), anti-Myc (Cell Signaling Technology Europe, B.V., ZA Leiden, The Netherlands and Thermo Fisher Scientific, Schwerte, Germany) and anti-JMY (Santa Cruz, Texas, USA). Alexa Fluor-conjugated and IRDye (800CW, 680RD) secondary antibodies were from Thermo Fisher Scientific or LI-COR Biosciences GmbH (Bad Homburg, Germany), respectively. F-actin was visualized with Atto 488 phalloidin (Sigma).

The Arp2/3 inhibitor CK-666 was purchased from Sigma, latrunculin B was from Merck KGaA (Darmstadt, Germany) and TGF-β1 was from Promokine. Arp3-specific siRNA (ON-TARGETplus mouse *Actr3* was purchased from GE Healthcare (Europe GmbH, Freiburg, Germany). JMY-specific siRNA was purchased from mwg Eurofins Genomics (Ebersberg, Germany).

### Transfections and MRTF-SRF activity assays

Cell culture and transfections of NIH 3 T3 were carried out as recently described [21]. Briefly, transient transfections of DNA or 30 pmol of Arp3-specific siRNA (ON-TARGETplus mouse *Actr3* (GE Healthcare)) were achieved according to the manufacturer’s instructions using X-tremeGENE 9 DNA transfection reagent (Roche, Mannheim, Germany) and Lipofectamine RNAiMAX reagent (Thermo Fisher Scientific), respectively. Prior to the experiments, cells were serum-starved in 0.5% (vol/vol) fetal calf serum (FCS) containing medium for 16–24 h. For protein extraction, cells were lysed in lysis buffer (50 mM Tris/HCl, pH 7.4, 150 mM NaCl, 2 mM EDTA, 1% (vol/vol) Triton X-100, 0.1% (vol/vol) SDS, Complete EDTA-free protease inhibitor cocktail (Roche)) and the protein content was determined with the Micro BCA (bicinchoninic acid) protein assay kit (Thermo Fisher Scientific).

For luciferase reporter assays, the Dual-Glo luciferase assay kit (Promega, Mannheim, Germany) was used as already described [21]. NIH 3 T3 cells were co-transfected with the WH2/V-containing constructs, the p3D.A-Luc firefly luciferase reporter plasmid and the pRL-TK *Renilla* Luciferase control plasmid. Where indicated, cells were pre-transfected with siRNA, serum stimulated with 15% (vol/vol) FCS (7 h) or treated with CK-666 (100 μM, 7 h), latrunculin B (0.5 μM to 1 μM, 7.5 h) or TGF-β1 (10 ng/μl, 21 h). Data shown are fold inductions of normalized firefly luciferase to *Renilla* luciferase activities. The remaining supernatants were immunoblotted to validate correct protein expression.

To analyze endogenous target gene transcription, 3 × 10^5^ NIH 3 T3 cells were transfected with 1 μg of the indicated constructs under serum-starved conditions. Total RNA isolation and first-strand cDNA synthesis from 500 ng RNA was carried out according to the manufacturer’s instructions using the RNeasy Mini Kit (Qiagen, Hilden, Germany) and the Verso cDNA synthesis kit (Thermo Fisher Scientific), respectively. qRT-PCR was performed as recently described [21].

### Immunofluorescence and microscopy

Immunofluorescence staining was performed as described previously [21]. Images were taken using an Apotome-containing Axio Observer.Z1 or an Axio Imager.M1 microscope (Zeiss, Jena, Germany) both with a 63x oil objective and a monochrome CCD camera (AxioCam MRm). Quantification was performed by assessing the subcellular localization of endogenous MRTF-A in 50 WH2/V-overexpressing cells in each of three independent experiments.

### Protein precipitation and immunoblotting

Co-immunoprecipitation was carried out as described previously [21]. Briefly, NIH 3 T3 cells were co-transfected with pEF-Flag-actin-WT and either pEF-myc-JMY variants or pEF-MRTF-A-f.l.-HA in a total of 5 μg cDNA. Following serum-starvation for 24 h, transfected cells were lysed in magnetic beads lysis buffer (50 mM Tris/HCl, pH 7.4, 150 mM NaCl, 1 mM EDTA, 1% (vol/vol) Triton X-100, Complete EDTA-free protease inhibitor cocktail). To analyze the binding of JMY mutants to Flag-actin-WT, cell lysates were incubated with anti-Flag M2 magnetic beads (Sigma). To analyze the effects of myc-tagged JMY variants on Flag-actin:MRTF-A complexes, cell lysates were incubated with 4.8 μg of purified MRTF-A (2–261) together with anti-Flag M2 magnetic beads. Isolated GST-JMY-*V*/VVVCA domains were expressed in *Escherichia coli* BL21 (DE3) Rosetta cells by standard techniques. Cells were harvested in magnetic beads lysis buffer, sonicated and cleared by centrifugation at 10,800 x *g* for 30 min. Lysates containing MRTF-A-f.l.-HA together with Flag-actin-WT were incubated with anti-Flag M2 magnetic beads (Sigma) and the indicated *E. coli* supernatants comprising 6 μg of full-length GST fusion protein. Precipitation was performed for 2 h at 4 °C under constant rotation followed by at least three washing steps. Binding was analyzed by immunoblotting as already described [21]. Detection and quantification of proteins were carried out with an Odyssey CLx system (LI-COR Biosciences GmbH) and the associated software as indicated.

### Statistical analysis

Data represent means with corresponding SEM including minimum three independent biological replicates. Statistical analysis was performed using an unpaired one or two sample student’s t-test where significance is indicated by * *p* ≤ 0.05, ** *p* ≤ 0.01, *** *p* < 0.001. Calculations were done with Microsoft Excel.

## Results

**Fig. 1:**
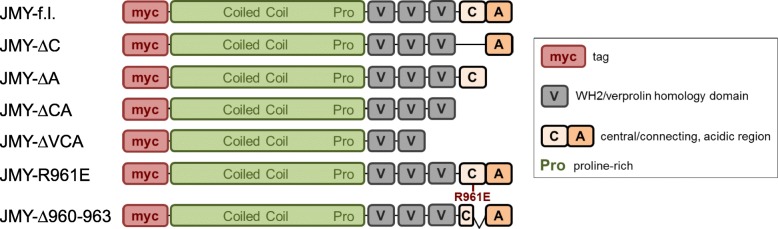
Schematic depiction of JMY constructs and domains used in this study

**Fig. 2:**
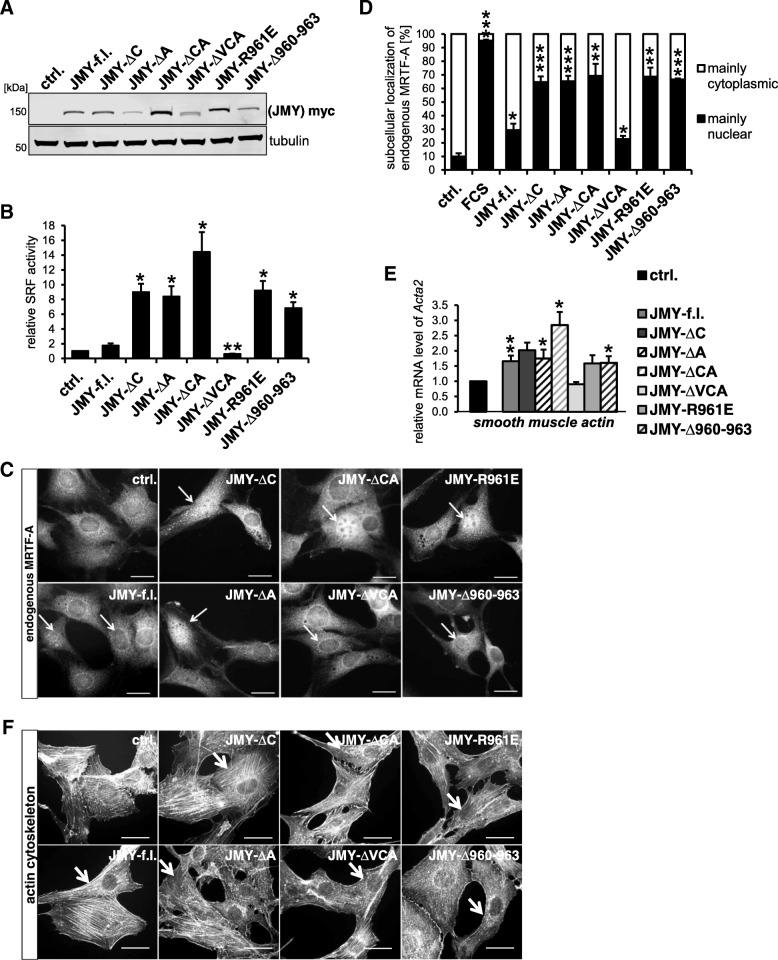
The C-terminal domain CA of JMY inhibits its ability of MRTF-SRF activation. The mouse fibroblast cell line NIH 3 T3 was transfected with the indicated JMY constructs and analyzed for MRTF-SRF activation under serum-starved conditions. **a**: Expression of myc-tagged JMY variants was controlled by immunoblotting with myc-specific antibody and tubulin. **b**: Relative MRTF-SRF luciferase reporter activity upon overexpression of myc-tagged JMY constructs. **c**: Nuclear translocation of endogenous MRTF-A in myc-JMY-overexpressing cells. Following serum starvation, cells were immuno-stained for endogenous MRTF-A localization. Arrows indicate myc-positive cells. Scale bars, 20 μm. **d**: Quantification of C by counting 50 myc-expressing cells for each of three independent experiments. **e**: MRTF-SRF-mediated transcription of smooth muscle α-actin (*Acta2*) in JMY-expressing cells. **f**: Organization of the actin cytoskeleton in myc-JMY-overexpressing cells. Following serum-starvation, cells were immunostained for endogenous actin. Arrows indicate myc-positive cells. Scale bars, 20 μm. All data were normalized to the serum-starved control which is set to 1 in **b** and **e**. Error bars, s.e.m., *n* = 3 (* *p* ≤ 0.05, ** *p* ≤ 0.01, *** *p* ≤ 0.001 according to an unpaired two sample student’s *t*-test (**d**) or an unpaired one sample student’s t-test (**b**, **e**))

The key regulatory step within the actin-MRTF-SRF pathway is the disruption of repressive G-actin:MRTF complexes. Besides activated actin polymerization, complex dissociation can be also induced by WH2/V-containing nucleation promoting factors such as N-WASP or WAVE2 [21]. Interestingly, JMY comprises three WH2/V domains and has dual nucleation as well as transactivation capabilities. Here, we analyzed whether JMY has a function in activating MRTF-SRF-mediated transcription beside its role as an actin nucleator. Therefore we generated myc-tagged JMY variants which were supposed to be deficient in binding and/or activating the Arp2/3 complex and expressed them in NIH 3 T3 mouse fibroblasts (Fig. 1 and 2a). The point mutant JMY-R961E as well as the deletion variant JMY-∆960–963 were designed according to the arginine residue 474 and the conserved KRSK (aa 473–476, mouse) motif of N-WASP which are crucial for Arp2/3 interaction [27, 28].

Overexpression of wild-type JMY resulted in a weak activation of a MRTF-SRF luciferase reporter in comparison to the serum-starved control (Fig. 2b). Interestingly, truncated JMY variants with deletions or a point mutation (R961E) within the central and acidic (CA) region exhibited a significant induction of MRTF-SRF reporter activity (Fig. 2b). The strongest MRTF activation was observed in JMY-ΔCA expressing cells. However, a JMY mutant lacking the VCA domain (JMY-ΔVCA) failed to induce the SRF reporter under these experimental conditions.

Induction of MRTF-SRF-mediated transcription frequently correlates with nuclear accumulation of MRTF-A. Therefore, we analyzed subcellular localization of endogenous MRTF-A in JMY-expressing cells. Overexpression of wild-type JMY induced nuclear accumulation of MRTF-A under serum-starved conditions which was further enhanced when the central and acidic region was mutated or deleted (Fig. 2c, d). In contrast, only a small fraction of cells expressing JMY-ΔVCA showed nuclear MRTF-A. Thus, SRF luciferase reporter activity correlated with the number of cells with nuclear MRTF-A.

To further validate these results, we examined whether JMY variants can activate transcription of the MRTF-SRF target gene smooth muscle α-actin (*Acta2*). Upon overexpression of wild-type or truncated JMY, the mRNA level of *Acta2* was found to be induced up to 1.5–2.5x under serum-starved conditions, whereas no effect was observed for JMY-ΔVCA (Fig. 2e). None of the constructs markedly perturbed the overall actin organisation in transfected cells stained with phalloidin (Fig. 2f). Together, these results suggest that the CA region of JMY may have an inhibitory role in activating MRTF-SRF-mediated transcription. Moreover, they demonstrate that MRTF-A is activated by JMY independently of its interaction with Arp2/3, whilst an intact WH2/V region seems to be required.

**Fig. 3:**
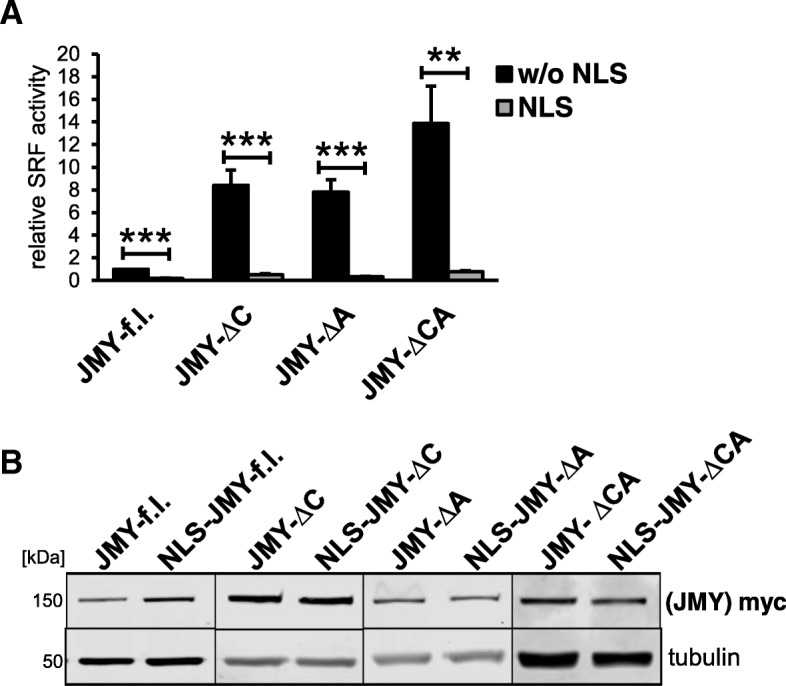
Reduced MRTF-SRF activation by JMY upon fusion to a nuclear localization signal (NLS). **a**: NIH 3 T3 mouse fibroblasts were transfected with the indicated JMY constructs, with and without a NLS fused to the N-terminus, and analyzed for MRTF-SRF activation under serum-starved conditions. **b**: Expression of myc-tagged (NLS-) JMY variants was controlled by immunoblotting with myc-specific antibody and tubulin as a control. Data are normalized to induction by JMY full length. Error bar*s*, s.e.m., *n* = 5 (* *p* ≤ 0.05, ** *p* ≤ 0.01, *** *p* ≤ 0.001 according to an unpaired two sample student’s t-test)

Because of JMY’s ability to shuttle into the nucleus and its role as a transcriptional coactivator, we next asked whether a forced nuclear presence of JMY differentially affects MRTF-SRF activity. Therefore we expressed JMY constructs fused to a nuclear localization signal (NLS). Compared to JMY without harboring an artificial NLS, such fusion proteins had a markedly reduced effect on the MRTF-SRF reporter (Fig. 3). Despite similar protein levels, hardly any MRTF activation was observable by NLS-JMY-ΔC, −ΔA or -ΔCA. This result excluded the possibility that nuclear accumulation of JMY is required for inducing MRTF-A, and rather suggested that JMY affects MRTF-SRF signaling via functions in the cytoplasm.

**Fig. 4:**
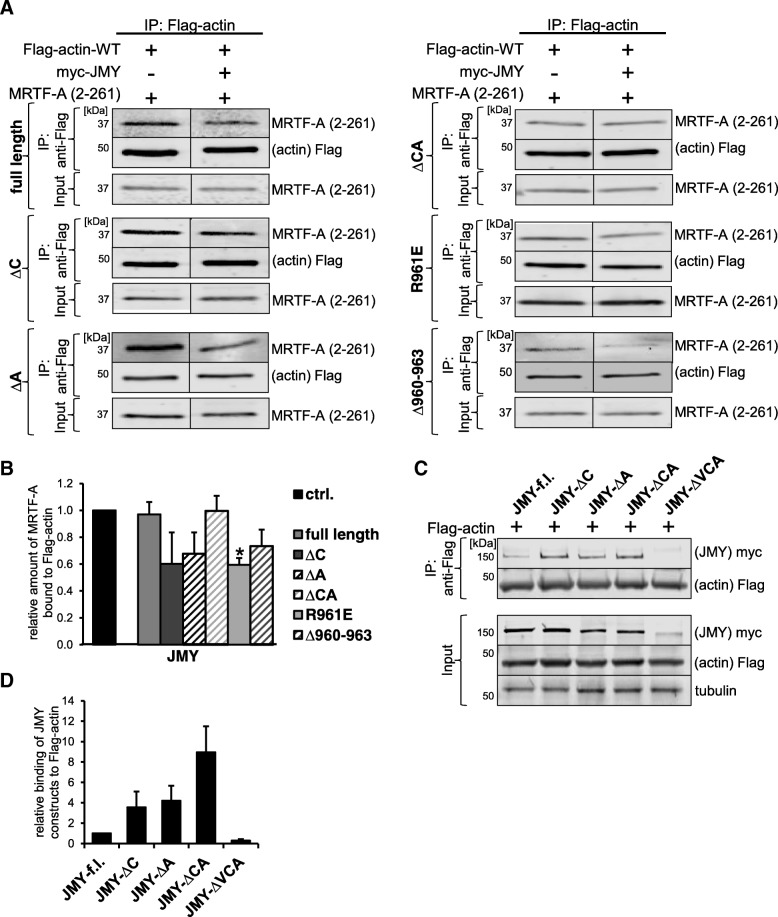
Truncated JMY competes with MRTF-A for actin binding. **a**: NIH 3 T3 cells co-expressing Flag-actin-WT and myc-JMY constructs were serum-starved for 24 h post transfection. **a**: Purified MRTF-A (2–261) was added to the lysates prior to immunoprecipitation with anti-Flag magnetic beads. Binding of proteins (IP panel) as well as the input control (Input panel) were detected with the indicated antibodies. Both lanes for MRTF-A (2–261) or Flag-actin-WT are from the same immunoblot, respectively. **b**: Quantification of precipitated MRTF-A (2–261) by Flag-actin upon co-expression of various JMY constructs. **c**: Immunoprecipitation with anti-Flag magnetic beads. Binding of proteins (IP panel) as well as the input control (Input panel) were detected with the indicated antibodies. Both lanes for myc-JMY constructs or Flag-actin-WT are from the same immunoblot, respectively. **d**: Quantification of precipitated JMY variants by Flag-actin. All data were normalized to myc-JMY-f.l. Error bars, s.e.m., *n* = 3

We recently showed that WH2/V domains as well as N-WASP and WAVE2 variants compete with MRTF-A for actin binding [21]. Therefore, we examined whether truncated JMY proteins can reduce the amount of MRTF-A bound to G-actin. To address this question, we assessed the binding of Flag-actin to the purified RPEL domain of MRTF-A (amino acids 2–261) upon co-expression with JMY variants. Mutating or deleting parts of the central and acidic region of JMY reduced the amount of actin:MRTF-A (2–261) complexes up to 30–40% in comparison to the control, in line with the SRF luciferase reporter assay (Fig. 4a, b). However, wild-type JMY and JMY-ΔCA failed to result in an observable reduction of MRTF-A binding to actin under these experimental conditions. Given that four constructs harboring deletions or mutations of the CA region increased the competitive potential of JMY, we conclude that the CA region restricts JMY for competing with MRTF-A for actin binding.

To assess the actin binding capabilities of the JMY constructs, we performed co-immunoprecipitations with Flag-actin. Wildtype JMY was coprecipitated with soluble Flag-actin, and deletion of the C-, A- or CA-region of JMY further enhanced its actin binding (Fig. 4c). In contrast, JMY-ΔVCA failed to show physical interaction. The quantification revealed a striking reminiscence between the actin binding of the JMY constructs and their effect on MRTF-SRF activation and nuclear localisation (Fig. 4d; compare to Fig. 2).

**Fig. 5:**
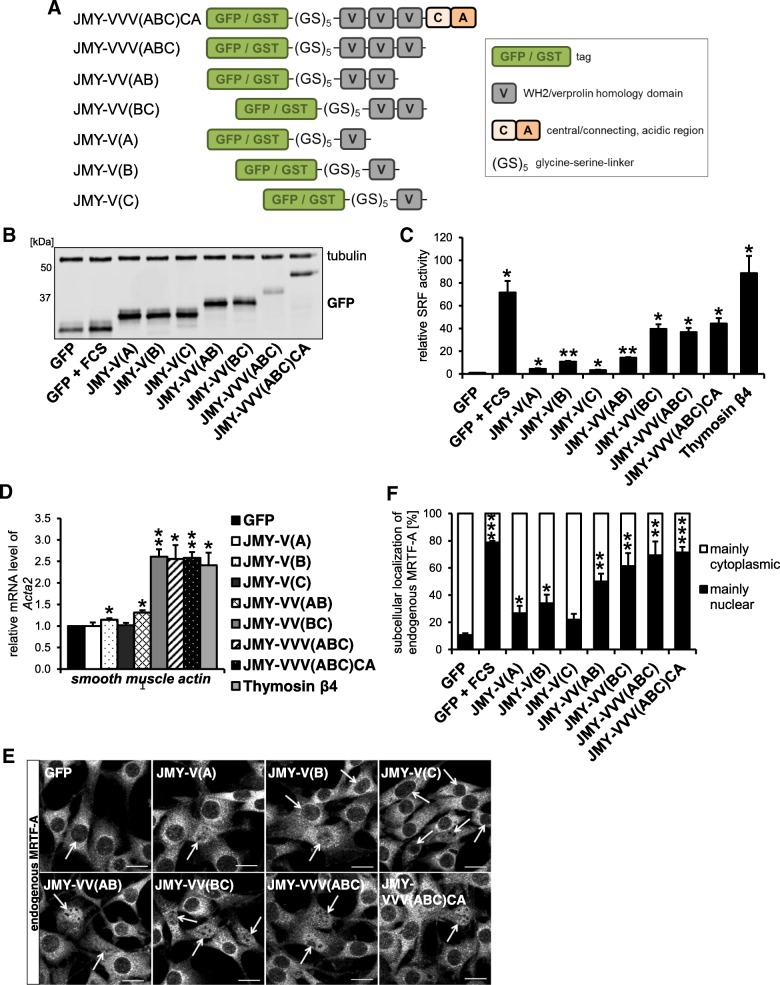
Isolated WH2/V domains of JMY activate MRTF-SRF-mediated transcription. Serum-starved NIH 3 T3 cells expressing the isolated V or VVVCA domains of JMY were analyzed for MRTF-SRF activation. **a**: Schematic overview of generated JMY-*V*/VVVCA constructs as GFP/GST fusion proteins with domain description. **b**: Overexpression of isolated JMY-V/VVVCAs detected by immunoblotting with a GFP-specific antibody. Tubulin was used as loading control. **c**: GFP-JMY-V/VVVCAs and Thymosin β4 induce SRF reporter activity compared to the starved GFP control (GFP). GFP-expressing control cells were stimulated with 15% serum for 7 h (GFP + FCS). **d**: Endogenous *Acta2* mRNA level in serum-starved NIH 3 T3 cells expressing the indicated JMY regions compared to the control (GFP). **e**: Subcellular localization of endogenous MRTF-A in GFP-JMY-V/VVVCA overexpressing cells. Arrows indicate GFP-positive cells. Scale bars, 20 μm. **f**: Quantification of E with 50 GFP-positive cells per construct in each of three independent experiments. All data were normalized to the serum-starved GFP control which is set to 1 in **c** and **d**. Error bars, s.e.m., *n* ≥ 3 (* *p* ≤ 0.05, ** *p* ≤ 0.01, *** *p* ≤ 0.001 according to an unpaired two sample student’s t-test (**f**) or an unpaired one sample student’s t-test (**c**, **d**))

To further narrow down the regions of JMY which are responsible for its regulatory role on MRTF-SRF activation, we generated fusion proteins which possessed different numbers and combinations of WH2/V domains as well as its complete C-terminal region. All regions were separated from the GST or GFP tags by a glycine-serine-linker (Fig. 5a). Overexpression of these constructs in NIH 3 T3 mouse fibroblasts significantly induced SRF reporter activity under serum-starved conditions, compared with the GFP negative control (Fig. 5b, c). Although activation of SRF by single WH2/V domains was generally weak, JMY-V(B) showed the strongest effect. Combining the first two WH2/V domains of JMY (JMY-VV(AB)) had little effect on SRF activity compared with JMY-V(B) alone, whereas JMY-VV(BC) and JMY-VVV(ABC) exhibited a profound induction of the SRF reporter. Interestingly, this activation was hardly increased in the presence of the Arp2/3-interacting CA region, suggesting that major effects on MRTF-SRF activity are regulated by WH2/V domains. As a positive control and in line with our previous findings, the WH2-only Thymosin β4 strongly activated the SRF reporter under serum-starved conditions [21, 29]. Moreover, the extent of MRTF-SRF target gene expression of *Acta2* by JMY-*V*/VVVCA fragments strongly correlated with their activating potential observed within the SRF reporter assay (Fig. 5c, d). In line with this, endogenous MRTF-A was found to be nuclear in at least 50% of cells expressing tandem WH2/V domains, whereas single WH2/V domains were less potent in inducing nuclear accumulation of MRTF-A (Fig. 5e, f). Together, these results indicate that JMY activates MRTF-SRF-mediated transcription via its WH2/V tandem repeats rather than by interacting with the Arp2/3 complex. However, considerable SRF activation requires at least two WH2/V domains, whereas JMY-VV(BC) showed the strongest effects.

**Fig. 6:**
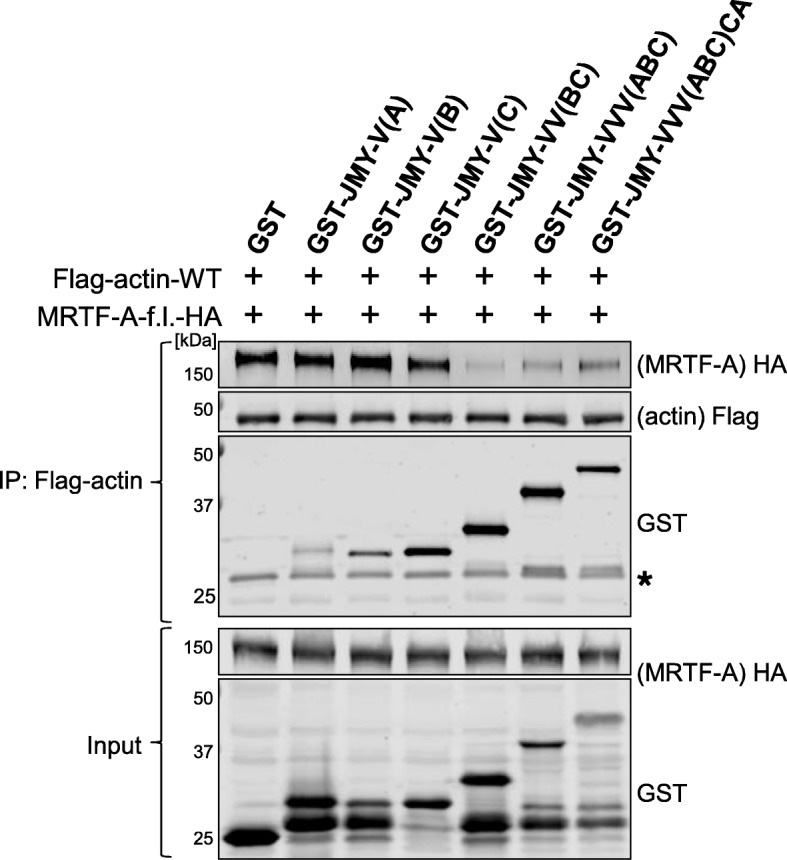
Recombinant V/VVVCA domains of JMY compete with actin for MRTF-A binding in vitro. Association of MRTF-A-f.l.-HA with Flag-actin-WT precipitates was investigated in the presence of cleared extracts of GST/-JMY-V/VVVCA-expressing *Escherichia coli* BL21 (DE3) Rosetta cells. Immuno-precipitates (upper panel) and lysate controls (lower panel) were analyzed by western blotting with the depicted primary antibodies. Asterisk indicates unspecific cross-reaction of GST antibody with *E. coli* proteins

We also tested whether isolated WH2/V fragments of JMY can compete with MRTF-A for actin binding. Therefore, we used lysates of JMY-*V*/VVVCA-expressing *Escherichia coli* cells. Extracts comprising the indicated fusion proteins were incubated with actin:MRTF-A complexes which were co-immunoprecipitated from fibroblast lysates, and the amounts of bound MRTF-A were determined by immunoblotting. Interestingly, tandem WH2/V domains as well as the whole C-terminal region of JMY strongly reduced the amount of MRTF-A bound to Flag-actin in comparison to the GST negative control (Fig. 6). Similar results were recently shown by using single WH2/V domains of WAVE2 and Spire2 [21]. In this experiment, however, isolated WH2/V domains of JMY failed to affect actin:MRTF complexes, potentially caused by a lower actin binding affinity (see Discussion). Despite technical limitations due to fusion protein disintegration and the failure to express JMY-VV(AB) in reasonable amounts, these results indicate that the WH2 region of JMY is sufficient to compete with MRTF-A for actin binding. Moreover, the apparent lack of MRTF-A reduction by JMY-ΔCA in Fig. 4a, b is inconsistent with the profound competitive effect by GST-JMY-VVV (ABC) seen here, suggesting technical issues in the negative outcome before.

**Fig. 7:**
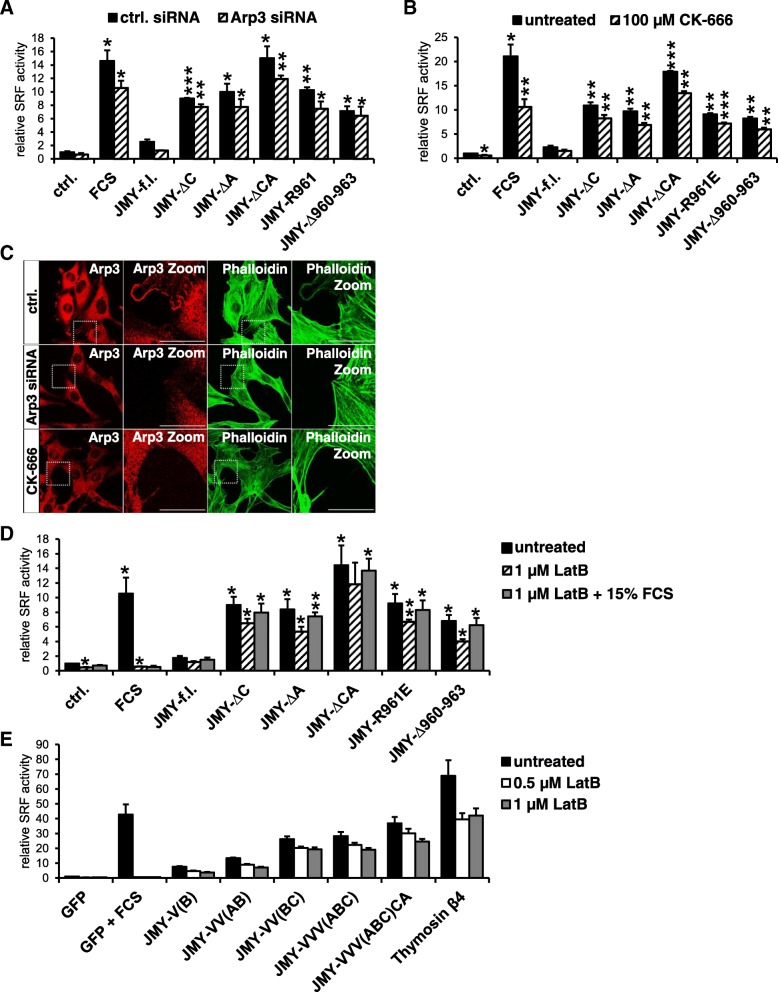
Truncated JMY variants and isolated JMY-V/VVVCA domains induce MRTF-SRF activity despite inhibited actin polymerization. MRTF-SRF activation by various JMY constructs was analyzed in serum-starved NIH 3 T3 cells following Arp2/3 inhibition or treatment with latrunculin B (LatB). **a**, **b**: Induced SRF reporter activity in myc-JMY overexpressing cells upon siRNA-mediated Arp3 knockdown (**a**) or treatment with 100 μM CK-666 for 7 h (**b**). As control, cells were stimulated with 15% serum for 7 h (FCS). **c**: Validation of Arp3 inhibition by immunofluorescence staining for endogenous Arp3 (red) and F-actin (Atto 488 phalloidin) (green). **d**, **e**: Significant induction of MRTF-SRF in JMY mutant (**d**) and JMY-V/VVVCA (**e**) cells following inhibited actin polymerization by LatB (0.5–1 μM, 7.5 h). All data were normalized to the starvation control which is set to 1. Error bars, s.e.m., *n* ≥ 3 (* *p* ≤ 0.05, ** *p* ≤ 0.01, *** *p* ≤ 0.001 according to an unpaired one sample student’s t-test)

To investigate whether MRTF-SRF activation by JMY is indeed caused by a direct competition rather than by induced actin polymerization, we next used Arp3-RNAi, the chemical Arp2/3 inhibitor CK-666 as well as the cytoskeleton drug latrunculin B (LatB). SiRNA-mediated Arp3 knockdown did not abolish serum- or JMY-induced MRTF-SRF activation (Fig. 7a). Similarly, JMY proteins activated SRF even upon inhibition of the Arp2/3 complex by CK-666 (Fig. 7b). Furthermore, C-terminal truncation or mutation of JMY still augmented the reporter activation. Basal SRF reporter activity also slightly decreased compared to control cells. Validation of Arp3 inhibition by RNAi and CK-666 was confirmed by absence of Arp3 from cellular protrusions and lamellipodial structures, and by western blotting following siRNA-mediated knockdown (Fig. 7c; and Ref. [21]). Together, these results show that MRTF-SRF activation by JMY is largely independent of Arp2/3-mediated actin polymerization. Furthermore, as wild-type JMY was still less effective compared with truncated JMY proteins, this indicates that the interaction with the Arp2/3 complex is not the cause for the lower activity of full-length JMY.

As JMY and its WH2 cluster were already shown to induce F-actin formation, we also analyzed the effect of the actin-depolymerizing drug latrunculin B on MRTF-SRF activation by JMY [5]. As expected, latrunculin B treatment strongly inhibited the activation of SRF by serum (Fig. 7d, e). Interestingly, overexpressed JMY still induced the SRF reporter even in the presence of latrunculin (Fig. 7d). Induction by wild-type JMY was considerably enhanced upon deletions or mutation of the central and acidic region, but largely unaffected by latrunculin. Furthermore, isolated WH2/V domains as well as the VVVCA region of JMY significantly activated SRF even though increasing amounts of latrunculin B were added (Fig. 7e). However, latrunculin B treatment generally lowered the induction of the SRF reporter. These results demonstrate that JMY activates MRTF-SRF at least partially independent of F-actin formation.

**Fig. 8:**
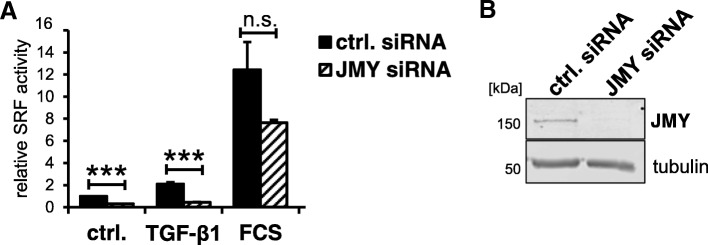
Depletion of JMY reduces MRTF activity **a**: NIH 3 T3 mouse fibroblasts were transfected with control siRNA and JMY-specific siRNA and analyzed for MRTF-SRF activation upon stimulation with TGF-β1 or FCS. **b**: Knock-down efficiency of JMY-specific siRNA was validated by immunoblotting with JMY-antibody and tubulin as a control. Data are normalized to siRNA control. Error bars, s.e.m., *n* = 3 (* *p* ≤ 0.05, ** *p* ≤ 0.01, *** *p* ≤ 0.001 according to an unpaired two sample student’s t-test)

Finally, we investigated the role of JMY on basal and induced SRF activity in fibroblasts. Knockdown of JMY by siRNA significantly reduced the MRTF-SRF activity in unstimulated control cells (Fig. 8a). Moreover, induction by TGF-β1 and serum was considerably decreased. The knockdown of JMY was validated by western blotting (Fig. 8b). These results suggest that, at least in fibroblasts, endogenous JMY is involved in transcriptional activity of MRTF and SRF. However, the stimuli and cell types critically requiring JMY for appropriate MRTF-SRF regulation await further characterisation.

## Discussion

JMY is a multifunctional protein which promotes F-actin nucleation by two distinct mechanisms. It recruits and activates the Arp2/3 complex via its CA region, similar to the WASP family of nucleation promoting factors [5]. Secondly, by virtue of three tandem WH2/V domains, it is capable of directly nucleating F-actin, similar to the actin nucleator Cobl [8, 16]. We show here that JMY activates MRTF-A mediated transcription independently of F-actin formation. Single, isolated WH2/V domains of JMY domains are sufficient for MRTF-A activation. Our data suggest that individual WH2/V domains are able to compete with MRTF-A for actin binding. As a consequence of elevated JMY binding to G-actin, the RPEL-mediated actin:MRTF-A complex dissociates, resulting in nuclear accumulation of MRTF-A and transcriptional activation of SRF target genes. Consistent with this, JMY-mediated MRTF-A activation was not associated with detectable alterations of the F-actin (Fig. 2f).

JMY’s independence from Arp2/3 for MRTF activation is evidenced by (i) deleting the C or the A region of JMY, (ii) mutating the residues (R961E and ∆960–963) homolog to those which were shown to be essential during WASP-mediated Arp2/3 induction [27], (iii) Arp3 knockdown and (iv) small-molecule inhibition of Arp2/3 function. In contrast, JMY constructs containing only one or two V domains are sufficient to (i) induce MRTF-A transcriptional activity, (ii) result in nuclear accumulation of MRTF-A, (iii) upregulate endogenous target genes and (iv) competitively inhibit actin:MRTF binding in pulldown assays (Figs 4 and 5). Finally, latrunculin B dramatically reduced serum induction of MRTF-A but did not diminish the impact of JMY constructs, excluding an important role of F-actin for JMY-induced MRTF-A activation. The association of latrunculin in the nucleotide binding pocket of G-actin alters the binding properties of the hydrophobic cleft, thereby enhancing repressive actin:MRTF complexes [12, 13, 26]. The activation of MRTF-A despite latrunculin treatment suggests a competitive binding of the WH2/V domains of JMY to latrunculin-laden actin.

However, not all V regions of JMY were equally effective for MRTF-A activation. Of the single WH2/V regions, the second domain V(B) showed the highest activation of the reporter, the endogenous target *Acta2*, and the nuclear accumulation. Similarly, VV(BC) was effective in MRTF activation and also dissociated the actin:MRTF-A complexes; the addition of the V(A) domain did not further enhance the effects observed. Thus we speculate that the second WH2/V domain of JMY harbors the highest competitive potential towards actin:MRTF complexes, and maybe the highest affinity for G-actin. Unexpectedly, however, JMY-∆VCA failed to increase the MRTF-SRF activity, despite the presence of VV(AB). Possible explanations include a missfolding problem of this particular JMY deletion construct, since appropriate domain length and borders were suggested to be crucial for WH2/V regions [30–32]. In line with this, JMY-ΔVCA was deficient for actin binding (Fig. 4c, d). The folding of the intrinsically disordered WH2/V domain onto G-actin might be influenced by neighboring residues, potentially also explaining why purified GST-V(B) did not compete for actin binding.

In line with the results presented here, we previously showed that N-WASP and WAVE2 activate MRTF-A partially by an Arp2/3 independent mechanism [21]. However, there is a striking difference in JMY: deletions or the R961E point mutation within the C-terminal CA region profoundly hyperactivated JMY and improved actin binding as compared to the wildtype full-length JMY. One possible explanation of our data includes an autoinhibition by e.g. intramolecular folding of JMY, similar to N-WASP. Alternatively, the alterations in the C-terminal portion may impair the formation of a repressive complex with an as yet unknown interaction partner. Similarly, JMY full-length was not efficient to dissociate actin:MRTF complexes unless the CA region was altered, suggesting an inhibiting role for the CA domain (Fig. 4). Since the activity of full-length JMY was suppressed compared with its isolated VCA domain, such an inhibitory mechanism was already previously proposed [33]. Consistent with this, deletions/mutations within the CA region induce an unfolded active state of JMY, resulting in the considerable SRF activation. Although Arp2/3 is known to bind to the CA region, the results argue against a role of Arp2/3 during autoinhibition, since its knockdown or inhibition by small molecules does not alter JMY activity towards MRTF. It remains to be investigated which amino acids, domains or accessory proteins are responsible for autoinhibition of full-length JMY.

JMY is implicated in a variety of cellular functions, including activation of p53 and p300 transcription factors, nuclear actin polymerization, formation of branched filaments at the leading edge, and cell motility [3–5]. Interestingly, its nuclear effects upon DNA damage are controlled by a regulated import mechanism very reminiscent to that of MRTF-A, by which JMY subcellular localization is controlled by mutually exclusive binding of G-actin and importin-α/β to an NLS within JMY’s WH2/V domains [16]. As JMY was shown to regulate nuclear events such as apoptosis, transcription and nuclear F-actin formation, it seemed possible that its competitive activation of MRTF-A is particularly important inside the nucleus. Our data suggest the opposite, however: JMY constructs with forced nuclear localization had only a minor effect on MRTF-A activity. The reason for this is unclear, considering that MRTF-A is known to respond to nuclear G-actin levels. One could speculate that the actin binding surface of JMY might be occluded by nuclear binding partners such as p300. Our result nevertheless indicates that most of the MRTF-A activation facilitated by JMY stems from a competition for cytoplasmic G-actin. Given that many MRTF-SRF target genes are supporting cell motility, this is consistent with the reported abrogation of the motility-promoting function of JMY upon nuclear accumulation [4]. More recently, JMY was implicated in vesicle trafficking and autophagosome assembly, adding more complexity to JMY function [24, 34, 35]. JMY remains a poorly understood member of the class I nucleation promoting factors, and data which connect the various cellular functions are scarce. An intricate crosstalk between JMY’s role at membranes, in the cytoplasm and in the nucleus is highly likely. However, it remains to be investigated whether activation of MRTF-A is integrated with some of the cytoplasmic roles of JMY, or antagonized by its nuclear functions.

## Conclusion

MRTF-SRF-mediated transcription is tightly dependent on the dissociation of the repressive G-actin:MRTF complex. The multifunctional JMY protein activates MRTF-A by dissociating the G-actin:MRTF complex in the cytoplasm, independently from its role in F-actin formation. Biochemical data indicate that the WH2/V domains compete with MRTF-A for G-actin binding. MRTF-A activation induced by JMY is negatively affected by its CA region, suggesting an autoinhibitory mechanism in full length JMY.

## Abbreviations

Arp: Actin-related protein
CA: Central/connecting, acidic
f.l.: Full-length
FCS: Fetal calf serum
IP: Immunoprecipitation
JMY: Junction-mediating and regulatory protein
LatB: Latrunculin B
MRTF-A: Myocardin-related transcription factor A
NLS: Nuclear localization signal
RPEL: Arginine-Proline-X-X-X-Glutamic acid-Leucine, actin binding motif
SEM: Standard error of the mean
siRNA: Small interfering RNA
SRF: Serum response factor
WH2/V: WASP/Verprolin homology 2
WT: Wild-type
